# The Low Molecular Weight Heparin Tinzaparin Attenuates Platelet Activation in Terms of Metastatic Niche Formation by Coagulation-Dependent and Independent Pathways

**DOI:** 10.3390/molecules23112753

**Published:** 2018-10-24

**Authors:** Lukas Maria Gockel, Jan Moritz Ponert, Svenja Schwarz, Martin Schlesinger, Gerd Bendas

**Affiliations:** Department of Pharmacy, University Bonn, An der Immenburg 4, 53121 Bonn, Germany; Lukas.Gockel@uni-bonn.de (L.M.G.); moritz.ponert@uni-bonn.de (J.M.P.); svenja.schwarz@uni-bonn.de (S.S.); martin.schlesinger@uni-bonn.de (M.S.)

**Keywords:** metastatic niche, heparin, platelet, thrombin, tumor, VEGF

## Abstract

An intimate interplay with platelets is an initial key issue for tumor cells in terms of hematogenous metastasis. Tumor cells activate platelets by different pathways and receive, upon forming a platelet cloak, protection from immune surveillance and support in metastatic niche creation. Therapeutic intervention with this early interaction is promising to antagonize the whole metastatic cascade. Here we aimed to investigate the capability of low molecular weight heparin (LMWH), unfractionated heparin (UFH), and a non-anticoagulant heparin derivative or FXa inhibitor fondaparinux to interfere with platelet activation by tumor cells. Coagulation-dependent and independent pathways of platelet activation by three tumor cell lines, and interference therewith were analyzed by fluorigenic thrombin formation assay, platelet aggregometry, ATP and VEGF release and endothelial tube formation assay. LMWH and UFH were found to repress various routes of platelet activation, reflected by attenuated endothelial tube formation. This confirms the duality of anti-coagulative and anti-adhesive properties of heparin. While non-anticoagulative heparin (RO-heparin) depressed platelets’ ATP and VEGF release by contact inhibition sufficiently, fondaparinux just attenuated tissue factor mediated thrombin generation. Concluding, these data suggest that LMWH as a guideline-based drug for anticoagulative strategies in oncology is promising to provide additional benefit for interference with metastatic activities.

## 1. Introduction

Venous thrombosis is a complication of cancer that is often accompanied by poor prognosis and reduced survival. Therefore, antithrombotic treatment is routinely applied to cancer patients. According to clinical guidelines for antithrombotic prophylaxis of cancer patients, low molecular weight heparin (LMWH) is the drug of first choice [1]. Besides the reduction of thromboembolic events, LMWH is also under debate to prolong patients’ survival by inhibition of tumor metastasis [2]. A positive effect of LMWH on attenuated cancer progression and survival has been observed in several clinical trials [3,4], whereas recent studies could not detect a significant survival benefit [5,6]. At the molecular level and beyond its anticoagulant activity, several mechanisms of LMWH have been elucidated, which potentially contribute to the interference with metastatic spread of tumors, namely inhibition of heparanase enzymatic and non-enzymatic activity or interference with adhesion receptor functions [7,8]. Several of these conducive effects of LMWH could be related to an inhibition of platelet-tumor cell communication. Tumor cells can activate platelets by different pathways, while this interaction with platelets conveys a multifaceted and crucial survival advantage to the tumor cells in the hematogenous dissemination phase [9,10]. Tumor cells instigate a platelet aggregation either by thrombin generation via tissue factor expression or by secretion of ADP or thromboxane A2, both of them induce a platelet activation via their receptor pathways [11,12]. Additionally, a direct contact between tumor cells and platelets has also been exhibited for platelet aggregation [13,14]. In consequence, platelets form a cloak around tumor cells immediately after tumor cells have invaded the blood circulation. This bears several advantages, for instance, cancer cells are protected from high shear forces in arterioles and from attack of natural killer (NK) cells [15,16,17]. Platelet derived chemokines recruit granulocytes to the tumor cell-platelet conjugates, which further support cancer cell survival [18]. Additionally, platelets are capable to shift cancer cell phenotype from an epithelial to a mesenchymal appearance by direct binding and secretion of transforming growth factor-β1 (TGF-β1) [19]. Those transformed cancer cells exhibit increased plasticity and invasiveness and feature stem cell-like traits [20]. Platelets are equipped with a large amount of different adhesion receptors on the cell membrane e.g., P-selectin, integrins and glycoproteins which finally modulate the arrest and adhesion of the platelet-tumor cell conjugates at the vascular wall [21,22]. Platelet secreted ATP or thromboxane A2 instigate endothelial cell retraction concomitant with an opening of the endothelial barrier and facilitated tumor cell extravasation [23,24,25]. Finally, platelet secreted growth factors like Vascular Endothelial Growth Factor (VEGF) contribute to angiogenesis in metastatic foci [26]. With a growing insight into the complexity of tumor cell-platelet communication, a therapeutic interference with the initial cancer cell mediated platelet activation appears promising and pivotal for the whole metastatic cascade. However, this should not solely be reflected by an antithrombotic approach. P-selectin has been considered as a crucial and primary target for heparin in antimetastatic approaches [27]. Nevertheless, it appears essential and promising to interfere with simultaneous inhibitory approaches, which could be represented by heparin. Heparin prevents thrombin formation and also blocks several adhesion receptors on platelet membranes, due to its sulfated polysaccharide structure [28,29,30].

In light of ongoing discussion concerning the first choice drugs in the anticoagulant field [31] and especially the antithrombotic prophylaxis of cancer patients, the role of LMWH has to be justified with the novel insights and postulated mechanisms of tumor cell-platelet communication and compared to other antithrombotic drugs, especially the direct oral antithrombotic drugs with respect to potential advantages resulting from its multiple functionalities and targets.

Here we aimed to simulate the different pathways of tumor cell-platelet communication, namely coagulation dependent and independent ones to investigate the capacity of LMWH to block platelet activation by tumor cells. To structurally relate the LMWH activities and illustrate the balance of thrombin dependent and independent activities, we compared LMWH or UFH with a non-anticoagulant heparin derivative and finally with the pentasaccharide fondaparinux as selective FXa inhibitor. Investigating various readouts of platelet activation and functional consequences, we provide evidence that LMWH and UFH represent a complex and more efficient interference with tumor cell induced platelet activation compared to solely FXa inhibition by fondaparinux or non-anticoagulant heparin functionality.

## 2. Results

### 2.1. Inhibition of Tumor Cell Induced Thrombin Generation

To investigate the coagulation-dependent pathway of platelet activation by tumor cells as target for therapeutic interference, we analyzed the thrombin generation capacity of tumor cells. Tissue factor (TF) expression by the tumor cells is considered as key step for initializing the coagulation cascade, therefore, tumor cells were selected in accordance to their TF expression. 

#### 2.1.1. TF Expression by Tumor Cell Lines

**Figure 1:**
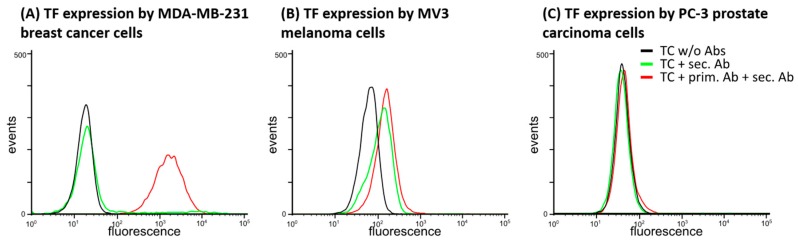
Flow cytometric analysis of TF expression by different tumor cells. (**A**) Human MDA-MB-231 breast cancer cells possess a strong TF expression. (**B**) Human MV3 melanoma cells display a certain capacity to express TF but they rank behind the indicated breast cancer cells. (**C**) The PC-3 prostate cancer cells exhibit nearly no fluorescence signals indicative of TF expression. Figures illustrate representative data of at least three identical experiments (*n* = 3).

We analyzed a series of tumor cell lines of different entities with respect to their TF expression capacity by flow cytometry, from which we finally selected three cell lines of various capacities. The triple-negative human breast cancer cell line MDA-MB-231, which is known as a highly aggressive and metastasizing one, displays a strong TF expression, as indicated in Figure 1A. The human melanoma cell line MV3, an accepted model cell for melanoma metastasis, has a clear capacity of TF expression, but this is evidently lower than in MDA-MB-231 cells (Figure 1B). Furthermore, we selected PC-3 human prostate cancer cells that displayed hardly any TF expression (Figure 1C) as a kind of negative control. With that selection of cells, which should represent differences in thrombin formation and platelet activation, we continued this study.

#### 2.1.2. Thrombin Generation and its Inhibition

**Figure 2:**
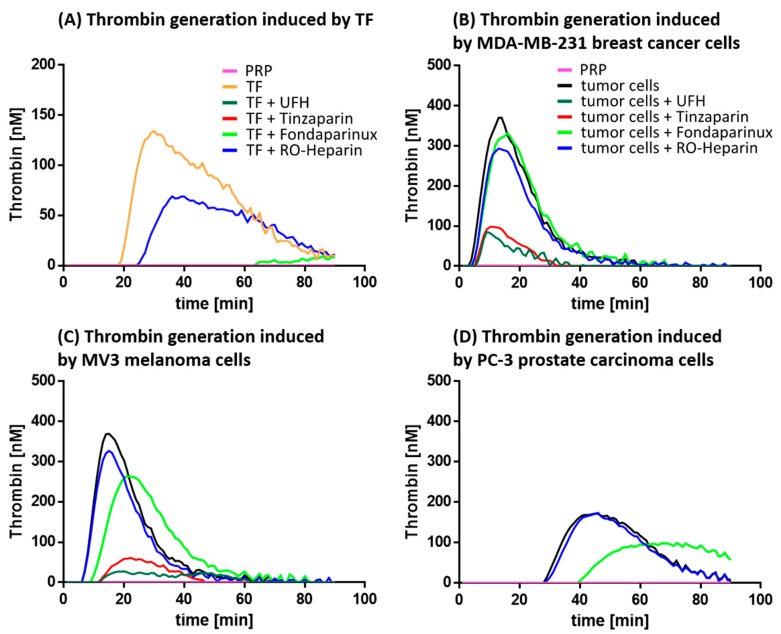
Thrombin generation by tumor cells and the interference by anticoagulants. (**A**) The addition of TF to the fluorigenic thrombin generation assay induces a signal that can be diminished by UFH, tinzaparin and fondaparinux, but not by the non-anticoagulant RO-heparin. Thrombin generation by (**B**) MDA-MB-231 cells, (**C**) MV3 melanoma cells, and (**D**) PC-3 prostate cancer cells and the inhibitory effects of the heparin derivatives, or fondaparinux, respectively. While UFH and tinzaparin prevent thrombin generation nearly completely in the individual approaches, and the inability of RO-heparin confirms the non-anticoagulant properties of this derivative, the restricted activity of fondaparinux remains elusive and probably refers to other activation pathways. The data are representative illustrations of at least three identical experiments.

To analyze the thrombin generation in PRP by the selected tumor cells, we applied a fluorigenic thrombin generation assay. To initially validate the function of this assay, which detects the kinetics of fluorescence increases resulting from thrombin protease activity, we checked the system in absence of tumor cells, and spicked the assay with recombinant TF. We also added corn trypsin inhibitor (CTI), which is known to prevent plasma coagulation by contact activation, to specifically emphasize TF-initiated coagulation. As indicated in Figure 2A, after a certain lag time of approximately 20 min a clear fluorescence peak appears, indicating a thrombin activity and the suitability of the assay. This thrombin generation can clearly be depressed to the baseline level by the addition of tinzaparin, UFH and fondaparinux, respectively, each taken at an adapted therapeutic concentration. In line with our expectations, RO-heparin, a non-anticoagulant heparin derivative described before [32], only slightly affects thrombin generation.

MDA-MB-231 cells induce a strong thrombin generation, indicated by the higher fluorescence signals and an earlier onset of the peak (Figure 2B) compared to the TF approach before. UFH and the LMWH tinzaparin, both at adapted therapeutic concentration, massively interfere with the thrombin generation dropping the signal to roughly one fifth of the approach without heparin. Again, RO-heparin is hardly able to interfere with thrombin formation and possesses only a slight down-shift of the curve. However, fondaparinux is also not effective in that approach. Reasons for that might be complex, probably the therapeutic concentration is not sufficient, but this appears not likely with respect to the efficiency that the pentasaccharide has shown before in the thrombin generation assay using TF (Figure 2A). Otherwise, despite excluding the intrinsic coagulation pathway by CTI, other activation routes circumventing FXa activities may occur. If so, these could more efficiently be interfered by a combined Xa and IIa inhibition mediated by heparin than by a pure Xa inhibitor.

The thrombin generation by MV3 melanoma cells (Figure 2C) is in principle identical to that of MDA-MB-231 cells, a slightly longer lag time before thrombin activity can be detected and corroborates the lower TF expression, shown before. Nevertheless, the excellent capacities of UFH and tinzaparin to block thrombin generation as well as the disability of RO-heparin support the above findings. Interestingly, fondaparinux displays a slight inhibitory effect inducing a right- and down-shift of the curve. The low-grade TF expressing PC-3 prostate cancer cells induce only a marginal thrombin generation kinetics indicated by the longer lag time and the curve height, which is reduced to baseline level by UFH and tinzaparin, not affected by RO-heparin, and slightly diminished by fondaparinux. For further statistical evaluations, the peaks of the thrombin generation curves were detected and statistically evaluated, as indicated in Supplementary Figure S1.

### 2.2. Coagulation Independent Readouts of Platelet Activation

In the following approaches, various experimental readouts of platelet activation were selected to focus on coagulation-independent pathways of platelet activation by tumor cells. 

#### 2.2.1. Interference with Platelet Aggregation

Platelet aggregation is a parameter of platelet activation measured in the clinical routine by light transmission aggregometry, where higher transmission correlates with stronger aggregation. In a recalcified platelet buffer, an impact of coagulation on platelet activation can be excluded. Consequently, activation should result from direct contacts between platelets and tumor cells and probably subsequent release of bioactive components for platelet receptor activation. 

**Figure 3:**
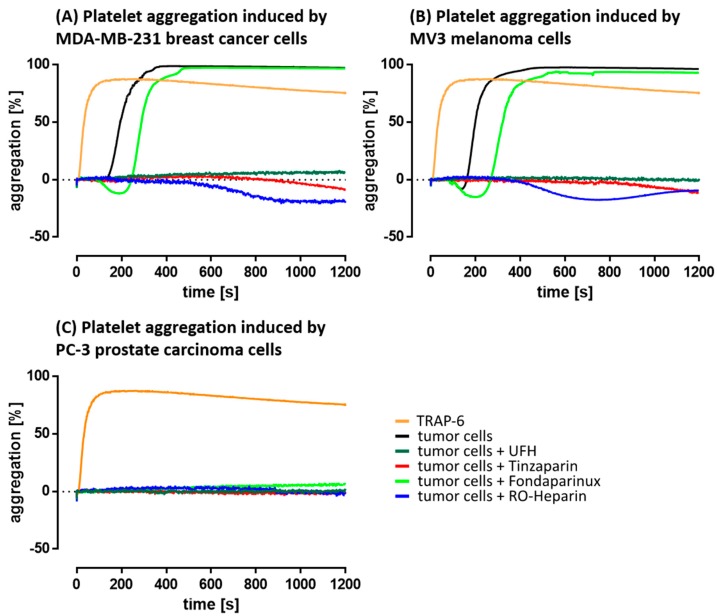
Aggregation of platelets as an activation parameter, measured by light transmission aggregometry in platelet buffer. TRAP-6 was applied as a functional control in the individual experiments (orange). (**A**) MDA-MB-231 cells trigger a massive platelet aggregation, which can be prevented by adapted therapeutic concentrations of UFH or tinzaparin, or RO-heparin (100 µg/mL). FXa inhibitor fondapariux is ineffective. Similar findings were obtained in the case of MV3 cells (**B**). (**C**) PC-3 cells have no impact on platelet aggregation. Data are representative of three identical experiments (*n* = 3).

As a functional control, the Thrombin Receptor Activating Peptide-6 (TRAP-6) induces fast and efficient platelet aggregation. MDA-MB-231 cells induce complete aggregation (Figure 3A) within a time frame of less than 400 sec. UFH, tinzaparin (each at an adapted therapeutic concentration), as well as the RO-heparin were able to depress the aggregation completely. Since FXa inhibition is not reflected by this assay due to the absence of clotting factors, the inability of fondaparinux at adapted therapeutic concentration to impact aggregation appears consequent. To further focus on a concentration-dependency of UFH, tinzaparin and fondaparinux for affecting platelet aggregation, a dilution series (UFH, tinzaparin) as well as higher concentrations (fondaparinux) have been applied (Supplementary Figure S2). Even higher concentrations of fondaparinux were without effect in this assay. 

Similar behavior of the anticoagulants became evident in case of MV3 melanoma cells (Figure 3B). Interestingly, PC-3 cells are not able to induce a platelet aggregation (Figure 3C) which illustrates that independent of the TF pathway, this cell line appears less functional to activate platelets also by other routes.

#### 2.2.2. Inhibition of ATP Release from Platelets

**Figure 4:**
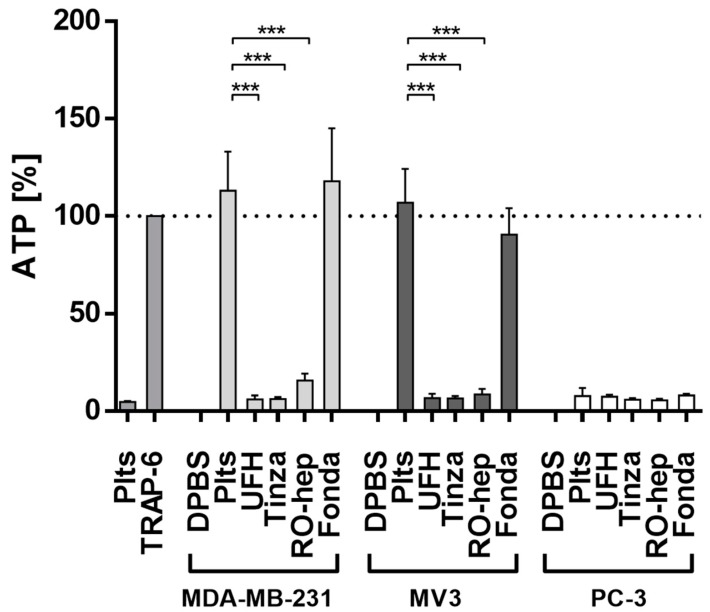
ATP release from platelets’ dense granules as indicator of activation, detected by luminescence measurements. The effect of TRAP-6 as a functional control was considered as 100% standard. MDA-MB-231 and MV3 cells induce a strong ATP release that can significantly be prevented by therapeutically relevant concentrations of UFH and tinzaparin; RO-heparin, while fondaparinux is ineffective. PC-3 cells display again no capacity to activate platelets (*n* = 3). Asterisks indicate statistical significance: *** *p* < 0.001.

The release of ATP from platelets’ dense granules is another functional and coagulation-independent parameter of platelet activation strongly contributing to metastasis, which was quantified by a luminescence measurement. Platelet secretion triggered by TRAP-6 via the thrombin pathway was selected as functional control and set as 100% (Figure 4, left). Platelet contact with MDA-MB-231 cells induces a massive ATP release, even stronger as in case of TRAP-6, which is significantly suppressed by UFH, tinzaparin, and RO-heparin. In contrast, fondaparinux is without any efficiency. Identical findings could be obtained in case of MV3 cells (Figure 4, middle), while PC-3 cells again fail in inducing a platelet activation (Figure 4, right).

#### 2.2.3. Inhibition of VEGF Release from Platelets and Functional Consequences

**Figure 5:**
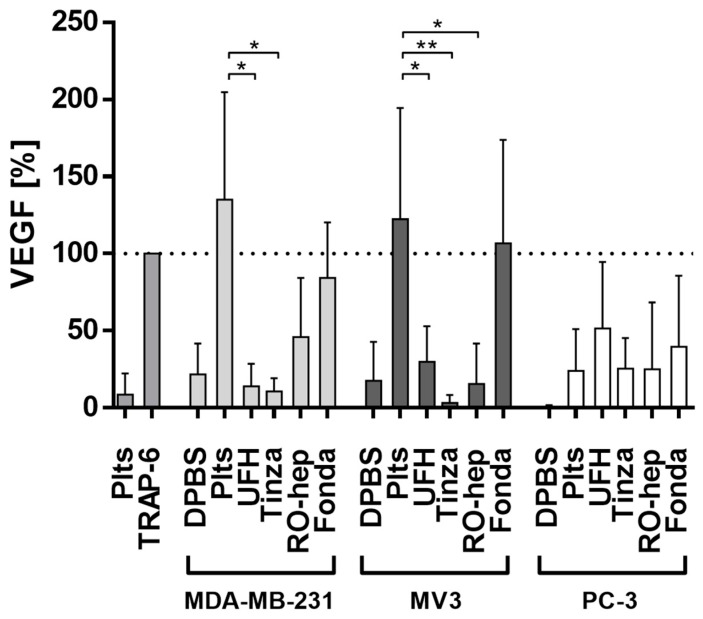
Detection of VEGF release from α-granules of platelets induced by tumor cells. MDA-MB-231 (left) and MV3 cells (middle) trigger a massive release of VEGF from α-granules, which can be significantly prevented by RO-heparin or therapeutic relevant concentrations of UFH or tinzaparin, while fondaparinux is unable to interfere with this activation route. Asterisks indicate statistical significance: * *p* < 0.05; ** *p* < 0.01.

The release of VEGF from platelets’ α-granules is considered as an essential step in forming the metastatic niche and to profoundly affect the endothelial cells to retract and thus lose barrier function, which enables the extravasation of tumor cells [33]. VEGF concentrations in platelet releasates induced by tumor cell contacts were determined by ELISA. While these determinations were in a coagulation independent background, TRAP-6 was again considered as a functional control and set as 100%. MDA-MB-231 and MV3 cells induce a distinct VEGF release, which is significantly suppressed by the three heparin derivatives (Figure 5). Fondaparinux is not able to interfere with VEGF release. The data obtained in the case of PC-3 cells appear arbitrary, these tumor cells hardly induce VEGF release, and in the presence of the anticoagulants a certain VEGF concentration is detectable, which should be related to individual deviations of the assay (Figure 5).

To reflect to functional consequences of VEGF release for endothelial cells angiogenic activation and a potential interference of the indicated anticoagulants, we performed a microscopic tube formation assay. Human EA.hy926 endothelial cells were seeded on a geltrex matrix, coincubated with platelet releasates and the number of branch points was evaluated microscopically after 24 h. The effect of TRAP-6 activated platelets was again considered as positive control and set as 100%. This was compared to the effect of added VEGF, which was nearly identical. Non-activated platelet releasates display a moderate low effect.

**Figure 6:**
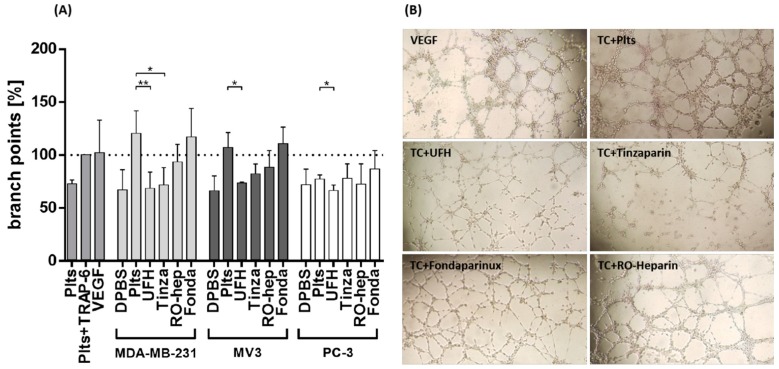
Tube formation assay to illustrate the impact of platelets, activated by tumor cells on the angiogenic activity of endothelial cells. (**A**) EA.hy926 endothelial cells were seeded on a geltrex matrix, coincubated with platelet releasates and the sprouts were quantified after 24 h microscopically. Releasates of platelets that have been activated by MDA-MB-231, MV3, or PC-3 cells are presented and the impact of the indicated anticoagulants at therapeutic concentrations thereon. UFH and tinzaparin possess significant effects to reverse the endothelial tube formation by MDA-MB-231 and MV3 cells treated platelets, while RO-heparin is less effective, fondaparinux is ineffective. (**B**) Representative images of the angiogenetic approach showing EA.hy926 cell cultivations triggered by VEGF or platelets releasates activated by MDA-MB-231 cells without inhibition “TC+Plt” or MDA-MB-231 cell activated platelet releasates after administration of the indicated anticoagulants. The tube formation density is evidently affected by UFH and tinzaparin. Asterisks indicate statistical significance: * *p* < 0.05; ** *p* < 0.01.

Releasates of platelet activated by MDA-MB-231 and MV3 cells cause a massive increase in branch points (Figure 6A), which can be significantly attenuated by UFH and tinzaparin in the MDA-MB-231 cells. RO-heparin has a visible effect to reduce the branch points in both cell approaches, while fondaparinux is without effects. In comparison to the other activation assay mentioned before, PC-3 cell induced platelet releasate displays no detectable impact on endothelial angiogenetic activation and sprout formation.

As an optical confirmation of these data, the microscopic images of endothelial tube formation by MDA-MB-231 cell-activated platelet releasates are given in Figure 6B. VEGF induces an evident sprouting of the EA.hy926 cells on the matrix. The tube forming activities are considerably attenuated when platelets have been pretreated with UFH and tinzaparin. The impact of RO-heparin and fondaparinux on blocking the sprout formation is evidently lower. Similar images for the behavior of MV3 cells as well as the behavior of PC-3 cells are indicated in Supplementary Figures S3 and S4, respectively. 

## 3. Discussion

In light of the increased risk of venous thromboembolism (VTE) of cancer patients compared with the general population, the optimal adjustment of cancer patients in terms of prophylaxis or treatment of VTE remains one of the great challenges in oncology. LMWH is presently the drug of first choice according to the therapeutic guidelines, which reflects, confirmed by the experience in applying these drugs for decades, their balanced safety profile and their superiority over vitamin K antagonists (VKA) in these terms. Although a clinical study confirmed that a six month treatment of cancer patients with acute symptomatic VTE, applying a daily therapeutic dose of tinzaparin resulted in a significant reduction of clinical nonmajor bleedings compared to warfarin [34], long term LMWH application has to be debated individually to consider potential risk factors, such as occurrence of HIT or coincidence with chemotherapeutic treatment causing neutropenia. With respect to safety aspects, the antidote situation for heparin therapy using protamine sulfate as the sole approved compound is not satisfactory with respect to inactivity towards LMWH and fondaparinux and the potential to cause severe adverse reactions in patients. However, recent efforts were reported to potentially replace protamine by other more effective heparin-binding copolymers [35,36,37], but these approaches are mainly at a preclinical level.

However, due to the general intention to shift from a parenteral route of drug application to an oral drug, many cancer patients were finally transferred to VKAs for longer treatments.

The trend for oral application becomes even more relevant considering the direct oral anticoagulants (DOACs), which have not yet been evaluated with respect to their therapeutic potential in oncology. A series of finalized or ongoing clinical trials comparing the VTE management in cancer by LMWH with DOACs requires justification of LMWH standing in oncology, considering potential effects and probably further benefits beyond anticoagulation [38,39,40]. On the basis of the interconnection of hemostasis with tumorigenicity and metastasis, LMWH has intensively been investigated in terms of potential antitumor efficiencies. A number of targeted activities, i.e. interaction with adhesion receptors, proinflammatory and tumorigenic signaling molecules or heparanase function have been confirmed [41]. Many of these studies were intended to illustrate the independence of these novel modes of action from the anticoagulant activities of heparin, and more related to the glycosaminoglycan (GAG) nature of heparin. Consequently, anticoagulation was often underestimated in these regards. However, arguments for novel targeted antitumor activities increasingly moved onto the defensive, since recent clinical trials of LMWH in oncology did not confirm a potential survival benefit of heparinized patients [5,6]. Reasons for this might be multifaceted, probably due to methodological issues, such as selection of tumor entities or treatment regimes, or because it is difficult to assess an aspired LMWH effect on survival in patients who are already receiving powerful cytotoxic treatments. More clinical trials will be necessary in the future to decide, whether heparin interferes with tumorigenicity. Likewise, a more detailed insight into molecular processes of metastasis appear essential to evaluate the need and balance of anticoagulant and other heparin effects in these terms and thus to distinguish from other anticoagulants.

The platelet activation by tumor cells, that we address here, appears as a meaningful issue for further consideration, since the crucial contribution of platelets to the metastatic progress of tumors is recognized and increasingly elucidated in molecular details [9]. We show that tumor cells activate platelets, from which they finally obtain versatile support and benefits for metastasis, by coagulation dependent and independent pathways. By selecting the indicated experimental conditions, the present *in vitro* approach allows a clear differentiation of both pathways, which will act in parallel *in vivo*. This emphasizes the model character of this study. LMWH possesses excellent capacities to interfere in this ambivalent scenario and a comparison with the other glycosidic drugs in this study illustrates that LMWH effects are based on its combination of GAG structure with anticoagulant activities. In contrast, solely an inhibition of FXa, represented by fondaparinux is not sufficient to interfere with the contact-induced pathways of platelet activation, while GAG without anticoagulation (RO-heparin) is deficient in thrombin pathway inhibition.

Our study provides evidence for the importance of contact induced platelet activation with respect to the subsequent secretion of granules, when applying a coagulation deficient background. The data suggest that platelets, once activated by contact stimuli, release the different types of granules simultaneously, since secretion of ATP from dense granules and VEGF from α-granules could be detected by the same activation triggers. We cannot totally exclude whether potentially an ADP release from the tumor cells contribute to platelet activation by binding P_2_Y_1_ and P_2_Y_12_ receptors, but the time scale of our experiments make this assumption unlikely. 

Concerning potential epitopes interacting at the tumor cell and platelet membranes, respectively, as targets for heparin interference, P-selectin appears as primary target. P-selectin is rapidly exposed to the platelet surface and has been confirmed as a key factor in hematogenous metastasis [22,27]. Heparin binding to P-selectin has been investigated comprehensively [42]. Notably, RO-heparin has been described as outstanding inhibitor of P-selectin [43], which corroborates the role of P-selectin in this experimental approach. In contrast, GPIIbIIIa at the platelet surface can rather be excluded as potential target. Although a heparin binding to this integrin, thus affecting its affinity to fibrinogen has been reported before [44], this seems to be less relevant in the present experimental context. Other potential candidates of mediating a contact-mediated activation, such as CLEC-2 or GPVI have not been associated with heparin binding yet and could be considered in further studies.

In summary, taking the tumor cell-platelet communication as an essential sub-process of the complex metastatic cascade of tumors, our model approach provides evidence that a sole anticoagulative therapeutic interference is not sufficient for a competent interruption of platelet activation and thus, subsequent metastatic activities. The combination of anticoagulant capacities of LMHW with potential antiadhesive properties is a unique feature and benefit in this regard, which should outmatch other conventional anticoagulants. This appears as a valuable argument for further LMWH application in oncology seeking antitumor activities. Regarding FXa inhibition and its evident inability to interfere in the indicated process, our study is limited to the evaluation of fondaparinux, which was selected with respect to the comparable glycosidic structure to heparin. Nevertheless, DOACs have to be considered in future studies to further confirm whether the indicated suitable bivalence in LMWH activity exceeds DOAC in a head-to-head comparison. 

## 4. Materials and Methods

### 4.1. Cell Lines

Human MV3 melanoma cells were cultivated in RPMI 1640 medium (PAN Biotech, Aidenbach, Germany) containing 10% (*v*/*v*) fetal calf serum (FCS) (Sigma Aldrich, Steinheim, Germany), 100 U/mL penicillin and 100 μg/mL streptomycin (PAN Biotech). EA.hy926 endothelial cells were cultured in Dulbecco’s modified Eagle’s Medium low glucose (DMEM low glucose) (Sigma Aldrich) with 10% FCS, 100 U/mL penicillin and 10 μg/mL streptomycin. MDA-MB-231 breast cancer cells were maintained in DMEM high glucose medium (PAN Biotech) and supplemented with 10% FCS, 1% l-glutamine (PAN Biotech), 100 U/mL penicillin and 100 μg/mL streptomycin. PC-3 prostate cancer cells were cultivated in RPMI 1640 medium containing 10% (*v*/*v*) fetal calf serum (FCS), 100 U/mL penicillin and 100 μg/mL streptomycin, 1% l-glutamine and 1% sodium pyruvate (Thermo Fisher Scientific, Waltham, MA, USA). All cells were incubated at 37 °C in a humidified atmosphere containing 5% (*v*/*v*) CO_2_. For subcultivation, MV3, PC-3, MDA-MB-231 and EA.hy926 cells were detached at a confluency of about 90% with trypsin/EDTA (5 g/L trypsin; 0.2 g/L EDTA × tetra sodium, Sigma Aldrich) for 5 min at 37 °C. Test for absence of mycoplasms were performed routinely every month.

### 4.2. Platelet Isolation and Activation

Platelets were isolated from one day expired human platelet-rich plasma (PRP) concentrates by centrifugation at 670× *g* for 10 min. The platelet pellet was resuspended in platelet buffer (10 mM N-2-hydroxyethylpiperazine-N9-2-ethanesulfonic acid, 140 mM NaCl, 3 mM KCl, 0.5 mM MgCl_2_, 5 mM NaHCO_3_, 10 mM glucose) and the platelet count was adjusted to 4 × 10^8^ platelets/mL. CaCl_2_ was added yielding a concentration of 1 mM. Prior to activation, platelets were preincubated with different anticoagulants for 30 min. The anticoagulant exposure was performed in platelet buffer using UFH (ratiopharm GmbH, Ulm, Germany) in a concentration of 1 I.U./mL, fondaparinux adjusted to a concentration of 775 ng/mL (Aspen Pharma, Dublin, Ireland), tinzaparin (LEO Pharma, Ballerup, Denmark) used in a concentration of 1 I.U./mL (corresp. roughly to 12 µg/mL) or reduced oxyheparin employed [45] in a concentration of 10 µg/mL, in aggregometry at 100 µg/mL. Applied concentrations of UFH, fondaparinux as well as tinzaparin were corresponded to therapeutic concentrations in anticoagulated patients or derived thereof, as indicated. All anticoagulants were diluted in water. Platelets were activated with 42.5 µM TRAP-6 (Tocris Bioscience, Bristol, UK) or 1 × 10^4^ tumor cells/mL for 12 min at 37 °C. Activation by tumor cells was carried out with MV3, PC-3 and MDA-MB-231 cells in presence and absence of an indicated anticoagulant. To obtain releasates, samples were centrifuged for 20 min at 1000× *g* and supernatants were collected. 

### 4.3. Flow Cytometric Detection of Tissue Factor Surface Expression

TF expression of tumor cells was investigated by flow cytometry. Cells were detached using an EDTA/DPBS solution. After centrifugation, the obtained pellet was resuspended in blocking buffer (3% BSA in DPBS) to a concentration of 1000 cells/µL and blocked for 15 min. Then, the wells were washed twice using a wash buffer (0.5% BSA, 0.1% sodium azide, DPBS). To label TF, the cells were incubated for 45 min with a primary anti-TF antibody (AF2339; R&D systems, Minneapolis, MN, USA) at a concentration of 2.5 µg/10^6^ cells. Subsequently, the samples were washed twice and incubated for 35 min with a FITC-conjugated, secondary antibody using 0.75 µg/10^6^ cells (SC-2777; Santa Cruz Biotechnology Inc., Santa Cruz, CA, USA). For each sample 10.000 cells were analyzed with a Guava easyCyte 3 HT reader (Merck Millipore, Billerica, MA, USA).

### 4.4. Thrombin Generation Assay

The tumor cell induced thrombin generation was evaluated using a fluorigenic thrombin generation assay. To eliminate effects of the intrinsic coagulation system, PRP was supplemented with Corn Trypsin Inhibitor (SC-204358; Santa Cruz Biotechnology). Afterwards, PRP was incubated with the indicated anticoagulant for 30 min and transferred to a 96-well plate. The plasma samples were spiked with DPBS and activated with tumor cells resulting in a final concentration of 1 × 10^4^ cells/mL PRP or with the TF containing reagent Technothrombin TGA RC high (Technoclone GmbH, Vienna, Austria). After adding the fluorigenic substrate reagent Technothrombin TGA SUB (Technoclone) the plasma samples were recalcified and thus the generation of thrombin was initiated. The kinetic of the subsequent, thrombin mediated Z-Gly-Gly-Arg-AMC cleavage was immediately measured by a plate reader (Fluoroskan Ascent, Thermo Fisher Scientific). Fluorescence signals were converted into thrombin concentrations using the evaluation software provided by Technoclone GmbH.

### 4.5. Light Transmission Aggregometry

Measurement of the coagulation independent platelet activation by tumor cells was performed by light transmission aggregometry (LTA) using an APACT 4004 aggregometer (Haemochrom Diagnostica GmbH, Essen, Germany). Therefore, platelets were isolated and handled as mentioned before. The activation was carried out at 37 °C in cuvettes with 1 × 10^4^ tumor cells/mL or 42.5 µM TRAP-6. Kinetics of aggregate formation was continuously measured by light transmission. As blank value (0% aggregation) non-activated platelets in platelet buffer and as positive control (100% aggregation) platelet buffer were employed.

### 4.6. ATP Assay

For the quantification of the platelets’ ATP release, an ATP determination kit was used according to the manufacturers’ instructions (Thermo Fisher Scientific). Platelets, tumor cells and anticoagulants were handled, coincubated and activated as aforementioned. Luminescence was measured by a Fluostar optima plate reader (BMG Labtech, Ortenberg, Germany).

### 4.7. ELISA

The VEGF concentrations of the platelet releasates were quantified using a human enzyme-linked immunosorbent assay according to the manufacturer’s instructions (VEGF; PeproTech Rocky Hill, NY, USA) and adapted to a viable dilution (4-fold) using an assay specific diluent. 

### 4.8. Tube Formation Assay

96-well plates were coated with geltrex (Thermo Fisher Scientific) for 30 min at 37 °C and 3.5 × 10^4^ EA.hy926 human endothelial cells together with platelet releasates were added per well. VEGF was added for positive control experiments. Tube formation was evaluated by branch point counting after 24 h of incubation. Representative areas were imaged with an Axiovert 200 microscope (Carl Zeiss, Jena, Germany). 

### 4.9. Statistical Analysis

Data represent means ± standard deviations of at least three independent experiments, if not indicated otherwise. Student’s *t*-test was applied for statistical analysis. *p* < 0.05 was considered statistically significant and marked with an asterisk. Two asterisks indicate a *p*-value below 0.01 and three stars were used for *p*-values below 0.001.

## 5. Conclusions

Our data provide an insight into the complexity of tumor cell-induced platelet activation as an essential step of hematogenous metastasis. Activation occurs by coagulation dependent and independent pathways, and LMWH has outstanding capacities to interfere with both routes going beyond anticoagulation, based on the unique GAG structure. This appears as a further argument for application of LMWH in oncological treatments referring to the ongoing discussion to search for the optimal VTE management of cancer patients. 

## Supplementary Materials

The supplementary materials are available online.
