# Supplementary material for: The Low Molecular Weight Heparin Tinzaparin Attenuates Platelet Activation in Terms of Metastatic Niche Formation by Coagulation-Dependent and Independent Pathways
Source: Molecules. 2018 Oct 24;23(11):2753. doi: 10.3390/molecules23112753 (PMC6278400; doi:10.3390/molecules23112753)
Supplement: Supplementary file 1 [file molecules-23-02753-s001.pdf]

Article

The Low Molecular Weight Heparin Tinzaparin Attenuates Platelet Activation in Terms of Metastatic Niche Formation by Coagulation-Dependent and Independent Pathways

Lukas Maria Gockel †, Jan Moritz Ponert †, Svenja Schwarz, Martin Schlesinger and Gerd Bendas *

Department of Pharmacy, University Bonn, An der Immenburg 4, 53121 Bonn, Germany; Lukas.Gockel@uni-bonn.de (L.M.G.); moritz.ponert@uni-bonn.de (J.M.P.); svenja.schwarz@uni-bonn.de (S.S.); martin.schlesinger@uni-bonn.de (M.S.)

† These authors contributed equally.

* Correspondence: gbendas@uni-bonn.de; Tel.: +49-228-735250

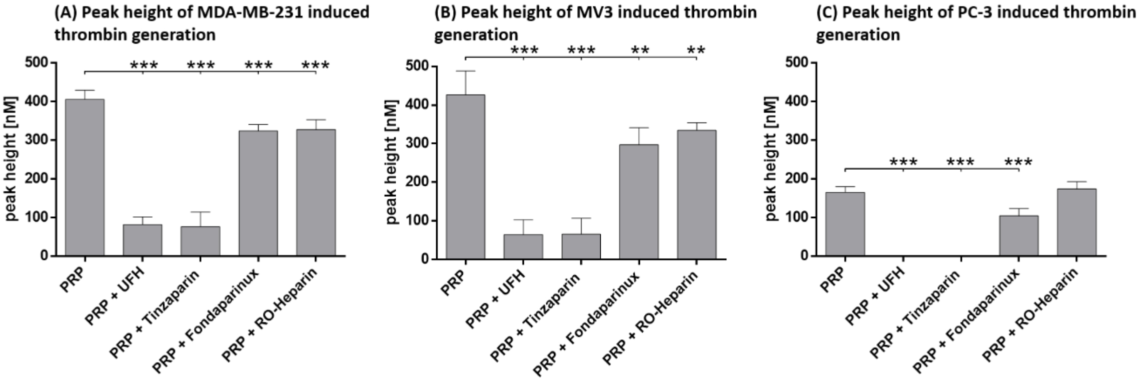

Figure S1. The inhibitory effect of the indicated anticoagulants on the thrombin generation due to a platelet preincubation with the tested compounds before contacting the tumor cells. The inhibitory effect is represented as the peak height of the thrombin generation kinetic curve, which was triggered by (**A**) MDA-MB-231 cells, (**B**) MV3 melanoma cells or (**C**) PC-3 prostate cancer cells, respectively. The figure illustrates the mean values of three identical experiments. Asterisks indicate statistical significance: *** p<0.001.

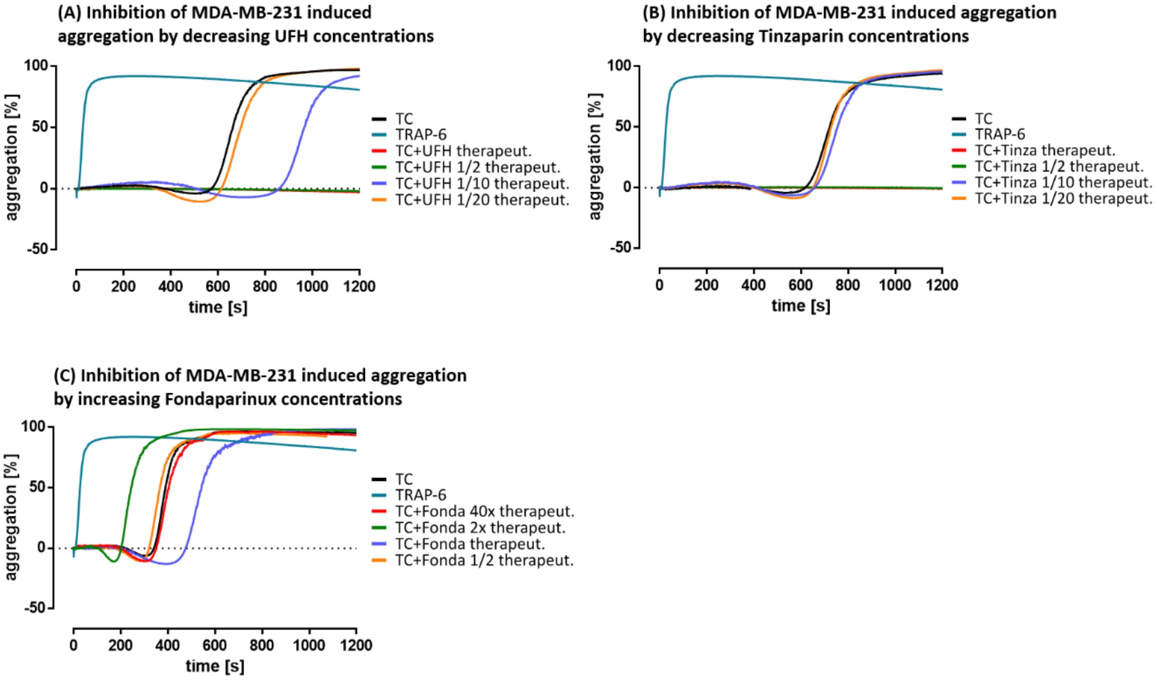

Figure S2. Since the adapted therapeutic concentrations of UFH and tinzaparin cause a complete inhibition of platelets aggregation, the impact of decreasing concentrations of (**A**) UFH and (**B**) tinzaparin were evaluated. For both anticoagulants, diluting the therapeutic concentration to one tenth or even more is associated with a loss to prevent MDA-MB-231 induced platelet aggregation. (**C**) To exclude a concentration dependency as a possible reason for the inability of fondaparinux to prevent platelets aggregation at the adapted therapeutic concentration, increasing concentrations were evaluated. Duplication of the therapeutic concentration or even higher concentrations (forty-fold) of fondaparinux could not cause an inhibitory effect.

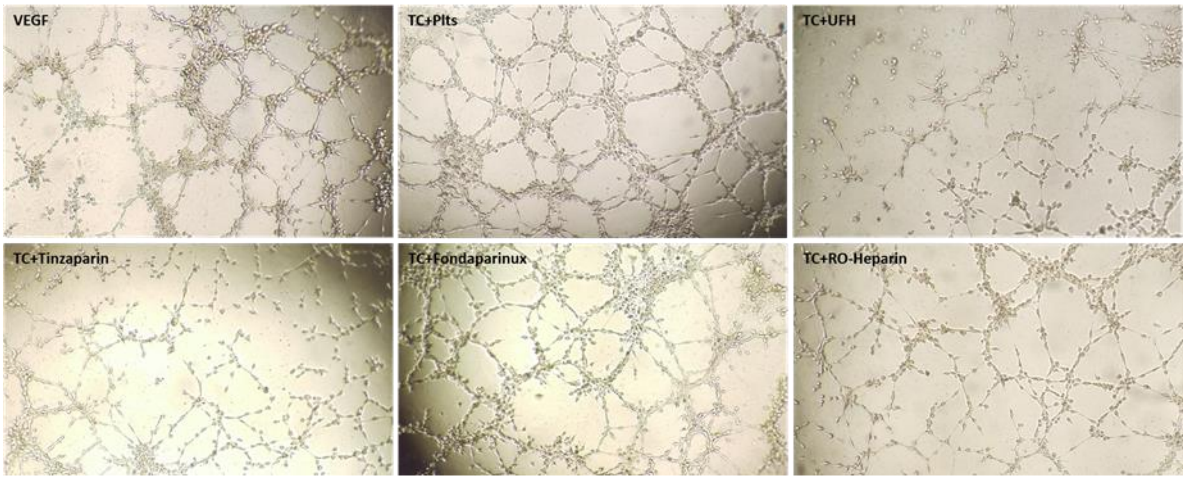

Figure S3. Tube formation assay of EA.hy926 endothelial cells on geltrex matrix and the impact of platelet releasates activated by MV3 melanoma cells and the inhibition by anticoagulants.

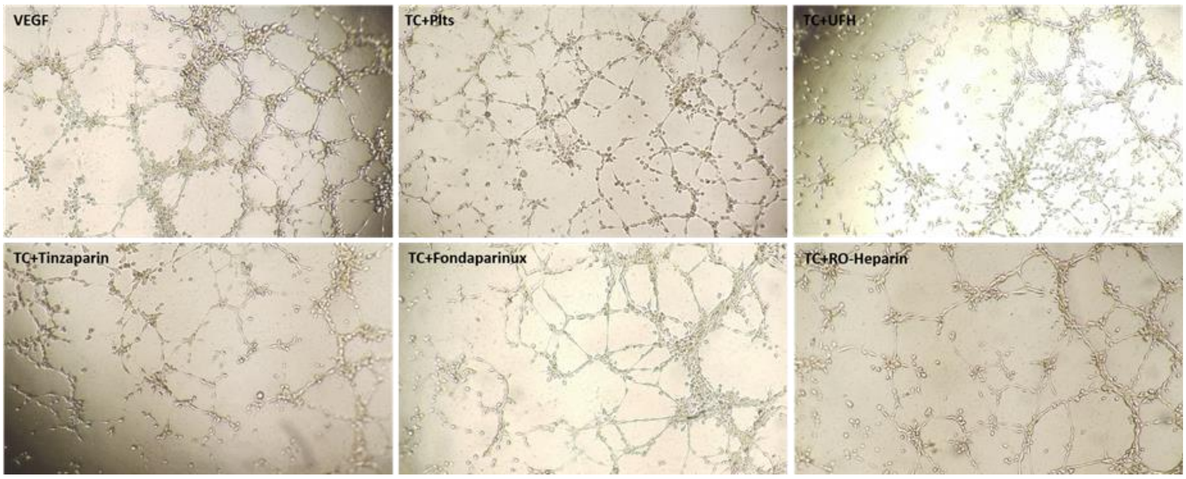

Figure S4. Tube formation assay of EA.hy926 endothelial cells on geltrex matrix and the impact of platelet releasates activated by PC-3 prostate cancer cells and the inhibition by anticoagulants.
